# Serine and one-carbon metabolisms bring new therapeutic venues in prostate cancer

**DOI:** 10.1007/s12672-021-00440-7

**Published:** 2021-10-27

**Authors:** Carlo Ganini, Ivano Amelio, Riccardo Bertolo, Eleonora Candi, Angela Cappello, Chiara Cipriani, Alessandro Mauriello, Carla Marani, Gerry Melino, Manuela Montanaro, Maria Emanuela Natale, Giuseppe Tisone, Yufang Shi, Ying Wang, Pierluigi Bove

**Affiliations:** 1 Department of Experimental Medicine, Torvergata Oncoscience Research Centre of Excellence, TOR, University of Rome Tor Vergata, a Montpellier 1, 00133 Rome, Italy; 2 San Carlo di Nancy Hospital, Rome, Italy; 3 IDI-IRCCS, Rome, Italy; 4 CAS Key Laboratory of Tissue Microenvironment and Tumor, Shanghai Institute of Nutrition and Health, Shanghai Institutes for Biological Sciences, University of Chinese Academy of Sciences, Chinese Academy of Sciences, 320 Yueyang Road, Shanghai, 200031 China; 5 The First Affiliated Hospital of Soochow University and State Key Laboratory of Radiation Medicine and Protection, Institutes for Translational Medicine, Soochow University, 199 Renai Road, Suzhou, 215123 Jiangsu China

**Keywords:** Serine, One-carbon metabolism, Prostate cancer metabolism

## Abstract

Serine and one-carbon unit metabolisms are essential biochemical pathways implicated in fundamental cellular functions such as proliferation, biosynthesis of important anabolic precursors and in general for the availability of methyl groups. These two distinct but interacting pathways are now becoming crucial in cancer, the de novo cytosolic serine pathway and the mitochondrial one-carbon metabolism. Apart from their role in physiological conditions, such as epithelial proliferation, the serine metabolism alterations are associated to several highly neoplastic proliferative pathologies. Accordingly, prostate cancer shows a deep rearrangement of its metabolism, driven by the dependency from the androgenic stimulus. Several new experimental evidence describes the role of a few of the enzymes involved in the serine metabolism in prostate cancer pathogenesis. The aim of this study is to analyze gene and protein expression data publicly available from large cancer specimens dataset, in order to further dissect the potential role of the abovementioned metabolism in the complex reshaping of the anabolic environment in this kind of neoplasm. The data suggest a potential role as biomarkers as well as in cancer therapy for the genes (and enzymes) belonging to the one-carbon metabolism in the context of prostatic cancer.

## One-carbon metabolism in cancer

Cancer cells can adapt their metabolism to achieve proliferation and survival benefits. The initial evidence for this observation can be attributed to Otto Warburg who demonstrated that cancer cells compared to normal cells increase aerobic glycolysis (Warburg effect) [1, 2]. In general, metabolic changes have been thoroughly studied and incorporated in the cancer hallmarks [3–5].

The involvement of one-carbon (1C) metabolism in cancer transformation has been just recently discovered getting intense interest in the last decades. The term “one-carbon metabolism” refers to a complex network of metabolic reactions, closely related and fundamental for cell energy production. The fulcrum is represented by folate metabolism [6]. 1C metabolism is essential to form, activate and transfer one-carbon units for different biosynthetic processes such us purine and thymidine synthesis and homocysteine remethylation [7].

The term folate indicates a series of molecules that share a common biochemical structure with three chemical moieties: a pteridine ring that can be reduced or oxidized, a para-aminobenzoic acid (PABA) linker that, together with the pteridine ring, binds 1C units and a variable chain length polyglutamate tail, that localizes the molecule within the cell. The biologically active form of folate is the reduced form tetrahydrofolate (THF); in humans, almost all natural folate species either taken from the diet or present in the body are in their reduced form of 5-methyl-THF [8].

**Fig. 1:**
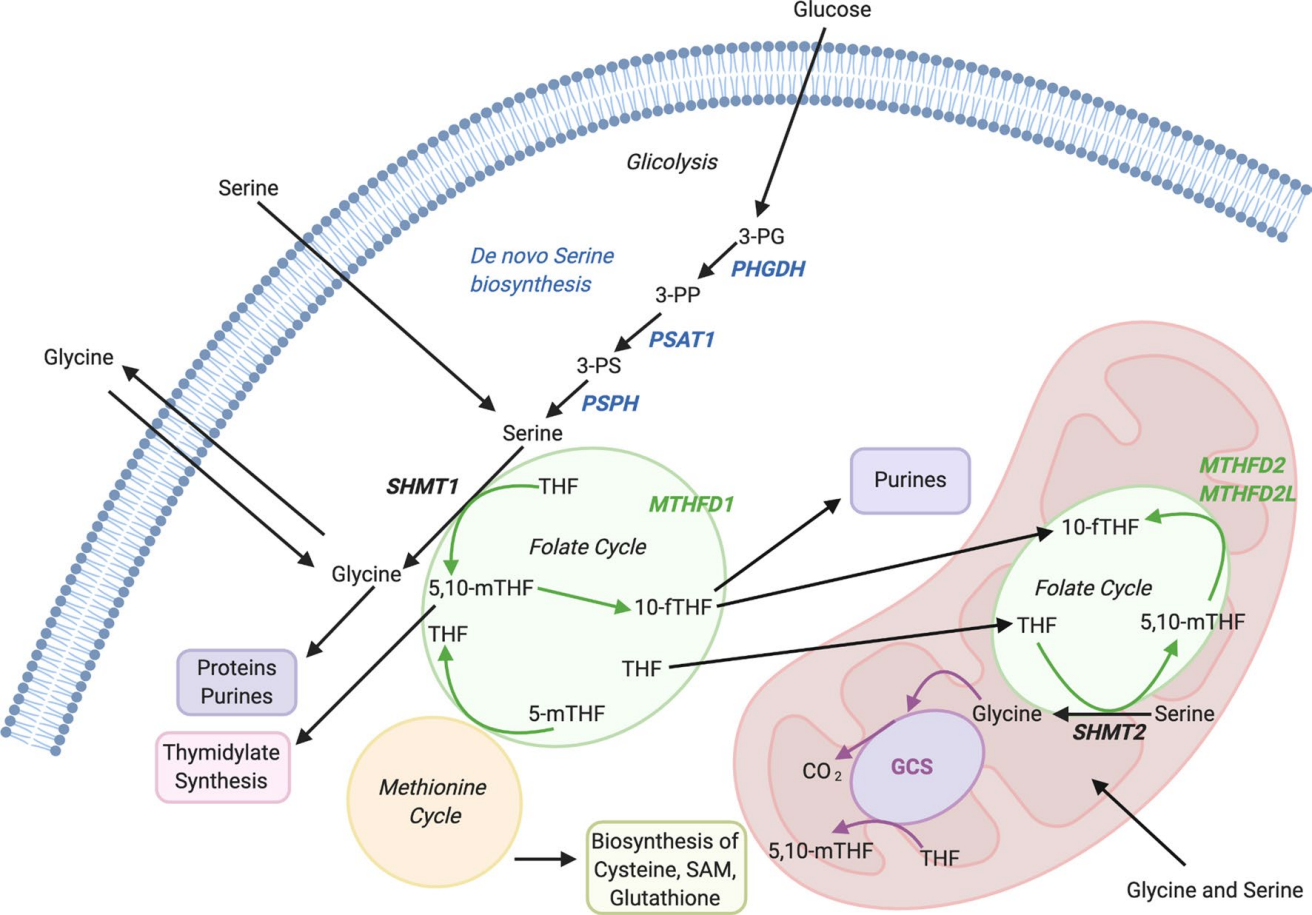
Schematic representation of the one-carbon metabolism. The schematic image describes biochemical pathway involved in serine and glycine biosynthesis and in one carbon metabolism, a group of reactions necessary to production of proteins, purines, thymidylate, methionine, cysteine, *S*-adenosyl-methionine (SAM) and glutathione. Regarding one-carbon units, the current image shows a strong metabolic connection between cytoplasmatic, and mitochondrial enzymes involved in THF production, serine de novo biosynthesis and serine and glycine catabolism. Cytoplasmatic enzymes: *PHGDH* phosphoglycerate dehydrogenase, *PSAT1* phosphoserine aminotransferase 1, *PSPH* phosphoserine phosphatase, *SHMT1* serine hydroxymethyltransferase 1; mitochondrial enzymes: *SHMT2* serine hydroxymethyltransferase 2, *MTHFD1* methylenetetrahydrofolate dehydrogenase1, *MTHFD2* methylenetetrahydrofolate dehydrogenase2, *MTHFD2L* methylenetetrahydrofolate dehydrogenase NADP+ dependent 2 like, *GCS* glycine cleavage system. Biochemical compounds: *3-PG* 3-phospoglycerate, *3-PP* 3-phosphopyruvate, *3-PS *3-phosposerine, *THF* tetrahydrofolate, *5,10-me-THF* 5,10-methylenetetrahydrofolate, *5-me-THF* 5-methylenetetrahydrofolate, *10-f-THF* 10-formate-tetrahydrofolate

1C metabolism can also been considered as an interlinked network of biosynthetic pathways, including serine metabolism, that allows the conversion of serine and tetrahydrofolate to glycine and 5′–10′ methylenetetrahydrofolate (5′–10′ meTHF), the glycine cleavage system (GCS) and the metabolism of choline and methionine (Fig. 1).

In general, this metabolic network allows cells to generate one-carbon units (methyl groups) and to utilize them for the biosynthesis of important anabolic precursors and for methylation reactions [9–11].

Several studies have highlighted an increased activation of two pathways that are involved in 1C metabolism in cancer: the de novo serine pathway and the mitochondrial 1C pathway [12, 13].

Consistent DNA synthesis is a fundamental request for cancer cells to sustain their proliferative capacity; therefore, multiple 1C metabolic enzymes are upregulated in cancer [14].

The importance of one-carbon metabolism in cancer was initially recognized in 1948, when Sydney Farber observed that dietary folate deficiency in children with acute leukemia reduced their leukemic cell number. Using the folic acid antagonist aminopterin in these patients it was possible to produce a temporary regression of the disease. These discoveries led to the development of the class of drugs known as antifolates [15].

One-carbon units are necessary for the biosynthesis of both purine and pyrimidine nucleotides, essential for DNA and RNA synthesis. Purine nucleotides are synthetized from ribose-5-phosphate, generated by the pentose phosphate pathway. Inosine monophosphate (IMP), the precursor to all purine nucleotides, is produced through a series of reaction, which require the incorporation of two one-carbon units and one molecule of glycine [16].

Depletion of serine, which is a substrate for glycine production, inhibits proliferation and reduces the level of purine nucleotides in cancer cells [17].

One-carbon units are also utilized to produce pyrimidine nucleotides and thymidylate. The thymidylate synthase (TYMS) enzyme can form dTMP from dUMP using methylene-THF as the methyl donor. Folate deficiency and methotrexate treatment can inhibit dTMP synthesis to such a degree that uracil is incorporated into DNA in its place [18, 19].

Cancer cells often show alterations in the patterns of DNA methylation and one-carbon metabolism represents the major source of methyl-group (one carbon units) utilized for DNA modification. DNA methylation regulates gene expression and hyper-methylation of promoters of tumor suppressor genes leads to a reduction in their expression. RNA is also subjected to methylation that can regulate translation. In addition, proteins can be post-translationally modified by methylation, which can alter their function or protein–protein interactions [20, 21].

*S*-Adenosylmethionine (SAM) is a universal methyl donor. Upon the transfer of its methyl group to an acceptor such as DNA, SAM becomes *S*-adenosylhomocysteine, which is converted to homocysteine. Homocysteine can be recycled back to methionine by the contribution of a methyl group from methyl-THF. Therefore, the primary role of serine is to support the methionine cycle to provide one-carbon units for methionine recycling through the reduction of THF to maintain the *S*-adenosylmethionine pool [1, 22, 23].

In addition, one-carbon metabolism plays a role in the production of NADH and NADPH, important cofactors that provide electrons for redox reactions. Enzymes that resynthesize THF can use NADH and NADPH as cofactors. As an example, during anabolism, the enzyme methylenetetrahydrofolate dehydrogenase (MTHFD) catalyzes the conversion of methylene-THF to formyl-THF for purine biosynthesis and NAD(P)+ is used as a cofactor and reduced to NAD(P)H in this reaction. The mitochondrial forms of this enzyme, MTHFD2 and MTHFD2L, can use either NAD+ or NADP+ as a cofactor, whereas the cytosolic form, MTHFD1, specifically uses NAD+ [24, 25].

Moreover, the 1C metabolism is also involved in the oxidative status homeostasis of the cell, since it is associated to the replenishments of glutathione through the pairing of the folate cycle and the methionine cycle [26–28] and in the immune/inflammatory response, so crucial during cancer progression [29–32]. This might also be ideal in the context of therapeutic intervention on the oxidative status of cancers in which the 1C metabolism is altered [33–35].

Serine and glycine are two fundamental amino acids that provide the precursors for the synthesis of proteins, nucleic acids, and lipids. Serine and glycine contribute to cellular metabolism by providing one-carbon units which refuels one-carbon metabolism in the cell [36–38].

This happens thanks to the presence of enzymes which can convert serine in glycine with one-carbon units formation; then glycine is further metabolized [37–39].

In eukaryotic cells, there are two different biosynthetic pathways producing serine: the de novo serine pathway and the serine recycling pathway, which is mediated by the Serine hydroxy-methyltransferases enzymes (SHMTs) and the folate cycle [40, 41].

Serine hydroxymethyl transferases are pyridoxal 5′-phosphate-(PLP) dependent enzyme that catalyze the reversible transfer of β carbon from l-serine to tetrahydrofolate (THF), with the sequent generation of glycine and 5,10-methylene-THF. This reaction represents the primary source of the one-carbon units required for the synthesis of thymidylate, purines, and methionine. Moreover, SHMTs in vitro are also able to catalyze THF-independent transamination, racemization, decarboxylation, condensation, and retroaldol cleavage reactions [42].

In some lung, ovarian and breast cancers SHMT1 acts as an oncoprotein, promoting tumor progression. The role of SHMT1 in hepatocellular carcinomas is still unclear, although some recent studies revealed a significantly decreased expression of SHMT1. This reduction is possibly correlated to adverse clinical features and poor prognosis of these cancer patients.

In several types of cancer an accentuated deregulation of SHMT2 is observed and is often associated with tumor progression. For example, in vitro studies of hepatocellular carcinoma cells show that inhibition of SHMT2 significantly decreases tumor progression while its overexpression is insufficient to induce tumor transformation, thus suggesting that SHMT2 is involved in tumorigenesis but it is not able to start malignant transformation alone. Anyhow, both the enzymes are somehow de-regulated by many modalities in the context of cancer [43–45].

## Serine metabolism in prostate cancer

In the pathogenesis of prostate cancer, the initial pathogenetic event might be considered the dependency from the androgen stimulation [46–50], but many more molecular alterations have been found associated to its progression [51–55] as well as to the development of specific clinical features of the disease such as bone metastasis [56] or studied as potential therapeutic targets [57]. The microbiome has also been shown to impact on the pathogenesis of this kind of cancer [58–62], also due to development of algorithms allowing to interpret its role in the context of a disease [63–67]. Anyhow, specific attention is focused on epigenetic remodeling.

DNA methylation has been associated to prostate cancer pathogenesis under different aspects such as in response to radiotherapy [68] or in the tracking of circulating tumor DNA [69] where androgen receptor sequences are hypomethylated in the blood stream and correlate with a poorer prognosis, suggesting also a possible role in liquid biopsies [70–75] and the overcome of the classical histology based biopsies [76, 77]. The methylation of the DNA strongly depends on the availability of methyl groups, by the activity of the enzymes involved in the 1C unit metabolism. An alteration of this metabolic pathway has therefore been explored, beneath partially, in the context of prostatic cancer.

The enzymes involved in the one-carbon metabolism have been evaluated in a prostate cancer population through genomic sequencing defining at least two methionine synthase gene (MTR) polymorphisms (rs2837281 T>G and rs1131450 G>A) associated to a poorer prognosis in the Han Chinese population [78]. Moreover, folate depletion has been shown to induce transcriptional reprogramming and to reshape DNA damage response and the DNA methylation pattern in a human xenograft model of prostate cancer following androgen withdrawal [79–82].

Anyhow, the one-carbon metabolism has also been implied in the pathogenesis of the highly resistant to treatment neuroendocrine histotype of prostatic cancer (NEPC) [83–86] since the de-regulation of protein kinase C (characteristic in NEPC) upregulates serine biosynthesis and SAM intracellular levels which allow the epigenetic reshaping of this peculiar type of cancer [87–89].

Prostate cancer has also been shown to depend on the activity of the methyladenosine phosphorylase enzyme (MTAP), the rate-limiting enzyme of the methionine cycle, which is used by prostatic cells to overcome the loss of carbon units in the secretion of polyamines. The dual inhibition on polyamines secretion and of MTAP has proved to induce apoptosis in ex-vivo explants [90].

Finally, the central component of the mitochondrial one-carbon metabolism MTHFD2 is upregulated in prostate cancer from at least two transcription factors ATF4 and c-Myc, which have already been implicated in this disease and its silencing inhibits prostate cancer cells growth as well as prostatosphere formation [91, 92].

Consistently with this preliminary evidence of an alteration of some of the one-carbon metabolism component in prostate cancer at different stages of the disease, we performed a bioinformatic analysis on publicly available gene expression and clinical data from the TCGA (Firehose Legacy) [93–95], comprehensive of 499 samples of prostate cancer specimens. The database has been explored using the cBioportal online platform [96, 97] and the GEPIA web server [98].

## Alterations of one-carbon and serine metabolism in a cohort of prostatic cancer patients from TCGA

**Fig. 2:**
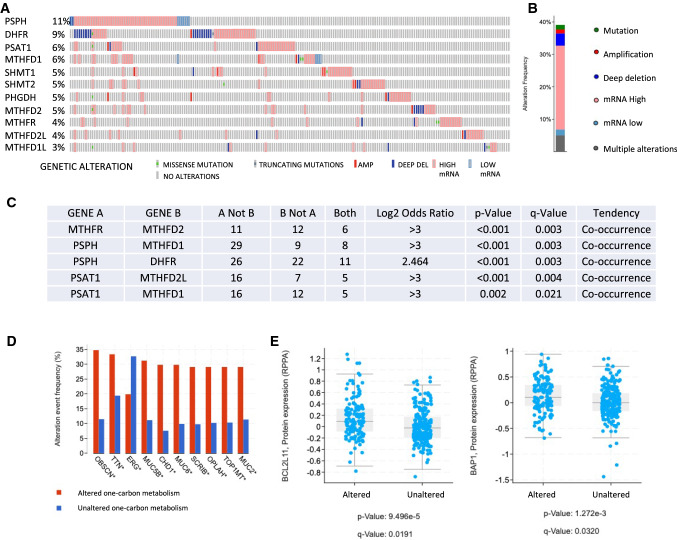
One-carbon metabolism enzymes in prostate cancer overview. **A** OncoPrint profiling of genetic or expression alteration in genes involved in the one-carbon metabolism in a cohort of 498 patients from TCGA (AMP, amplification, DEL, deletion); **B** cumulative alteration frequency of the one-carbon enzymes analysed; **C** co-occurrence analysis of the alterations of transcription the one-carbon enzymes genes, p-values are derived from one-sided Fisher Exact Test, q-values are derived from Benjamini–Hochberg FDR correction procedure; **D** genomic alteration frequency of known prostate cancer driver genes in one-carbon metabolism genes altered group vs control; **E** log2 scale protein expression of BCL2L11 and BAP1 in one-carbon metabolism genes altered group vs control, p-values are calculated from Student’s T-test, q-values are calculated from Benjamini–Hochberg procedure

The analysis of the expression of genes involved in serine and glycine metabolism has been evaluated (Fig. 2A). Phosphoserine phosphatase (PSPH), whose role in lung cancer has already been explored [99], shows a relatively high percentage of alteration in the prostate cancer cohort, up to 11% of the specimens, mostly due to gene overexpression (except for a few cases in which the gene is deleted). Dehydrofolate reductase (DHFR) [100] is the second most altered gene in the 9% of all cases. Differently form PSPH the DHFR gene does not have a clear pattern of alteration, showing gene overexpression in almost half of the cases in which the alteration is present, followed by downregulation. SHMT1 and SHMT2 [25, 101, 102] enzymes show altered expression in 5% of the cases, comparably to the other genes which are essentially involved in the folate cycle. From this analysis it is evident that in prostate cancer samples in which there is an alteration in one of the genes related to the one-carbon metabolism, it is quite probable that other genes from the pathway are altered so that it is unlikely that a single gene is the only one whose expression is perturbed. This might suggest that the reshape of the serine metabolism in prostate cancer is a pervasive metabolic event that is in place to adapt the transformed cell to gain selective advantage.

As a matter of fact, all together these genes are altered in nearly 40% of cancer specimens and most of the alterations of the genes involved in serine and glycine metabolism are due to increased expression of the relative mRNA rather that due to mutation. This evidence might still suggest that the serine and glycine metabolism is more an adaptive event, rather than a driver genetic aberration of the cancer itself (Fig. 2B).

As shown on Fig. 2A, there is a tendency to find more than one gene from the one-carbon metabolism altered in a single prostate cancer. This evidence can be further dissected showing that many genes have a statistically significative co-occurrence trend: methylene-tetrahydro-folate reductase MTHFR) is altered in samples in which also the MTHFD2 gene is altered (p < 0.001, q = 0.003), while PSPH overexpression is associated to MTHFD1 and DHFR (p < 0.001, q = 0.003 for both), which is also the case for PSAT1 and MTHFD2L (p < 0.001, q = 0.003), or with MTHFD1 (p < 0.001, q = 0.004) (Fig. 2C).

Consistently with the role played by the one-carbon metabolism in providing carbon units which are partially used to synthesize *S*-adenosyl-methionine, and its effect on the epigenetic remodeling of the chromatin, it is quite interesting to observe that in the serine metabolism altered group of patients (comprising all the patients showing one alteration in at least one of the genes shown in Fig. 1A) there is a relatively higher alteration (considering any kind of event, form mRNA over- or down-expression, to mutations, etc.) in many genes which are considered cancer drivers in prostate cancer, such as obscurin (OBSCN) [37], titin (TTN) [103], the ETS transcription factor (ERG) [104] and many mucins (MUC5B, MUC6, MUC2) [105, 106] (Fig. 2D). Alteration of the serine metabolism might therefore show promising therapeutic approaches if its role in epigenetic remodeling of prostate cancer is confirmed.

Serine metabolism reshaping in prostate cancer might also plays a rather pervasive rearrangement of cellular metabolism and expression profile and might be associated to changes of the expression of many more genes which have been clearly associated to tumor genesis and progression such as the apoptotic protein BCL2L11 [107–111] (more expressed in the group of patients harboring a perturbation of the serine metabolism, p < 0.001 and q < 0.001) and well as the chromatin remodeler BAP1 [112] (less expressed in the altered groups, p < 0.001 q = 0.003) (Fig. 2E).

## Mutational status and copy number alterations of one-carbon metabolism enzymes in prostate cancer patients

**Fig. 3:**
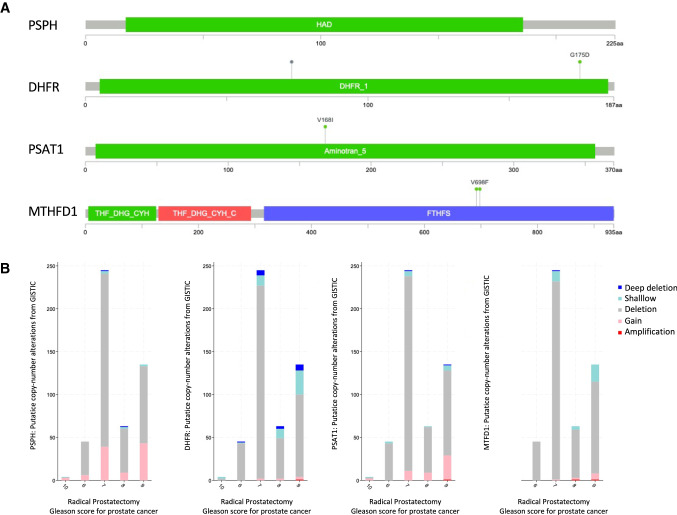
One-carbon metabolism enzymes mutations and correlation with Gleason score. **A** Schematic representation of the top four altered one-carbon metabolism genes and mutations in the TCGA analysed cohort; **B** putative copy-number alterations number of samples over the total 499 samples analysed in the TCGA Firehose Legacy cohort, of the top four altered one-carbon metabolism genes in groups of prostate cancer patients stratified according to Gleason score after radical prostatectomy

As said initially, genes in the serine metabolism are rarely mutated in prostate cancer. The most reported mutations are for the PSPH gene, with a mutation in its HAD domain, in DHFR, in PSAT1 and in the FTHFS domain of the MTHFD1 gene (Fig. 3A).

Since the genes involved in the serine metabolism are mostly subject to expression alteration, an analysis on the putative copy-number alteration form GISTIC shows a relatively increasing cumulative alteration in cancer specimens with higher Gleason scores (> 7), except for Gleason 10 cases which are just less abundant among all the cases and show quite a substantial number of samples harboring copy-number modifications (Fig. 3B).

**Fig. 4:**
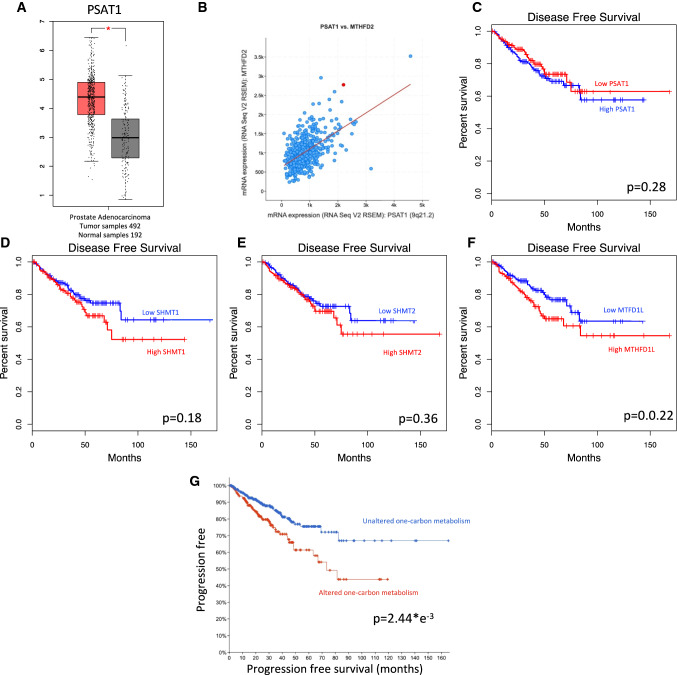
One-carbon metabolism enzymes clinical correlations. **A** PSAT1 log scale differential gene expression between prostate cancer tissues compared to normal matching controls (GEPIA), p-value is calculated applying the one-way ANOVA statistical method; **B** mRNA expression correlation of PSAT1 and MTHFD2 enzymes in prostate cancer tissues, Spearman coefficient 0.48 (p < 0.001, two-sided T-test) Pearson coefficient 0.54 (p < 0.001, two-sided T-test); **C**–**F** disease-free survival Kaplan–Meier plots stratifying patients according to expression levels of PSAT1 (**C**), SHMT1 (**D**), SHMT2 (**E**) and MTHFD1 (**F**) (GEPIA), p-values are calculated applying the log-rank test; **G** cumulative one-carbon metabolism alterations and progression-free survival of prostate cancer patients from TCGA (cBioportal), p-values calculated using the Logrank test

Some of the genes involved in the serine metabolism are differentially expressed between prostate cancer tissue and the surrounding normal prostate epithelium as in the case of PSAT1, overexpressed in the tumor (p < 0.005) (Fig. 4A). PSAT1 expression also shows a strong direct positive correlation with the expression of MTHFD2 gene with Spearman coefficient = 0.48 (p < 0.001) and Pearson coefficient = 0.54 (p < 0.001) (Fig. 4B).

## One-carbon and serine metabolism impact on prostate cancer progression

The progression of prostate cancer involves several molecular mechanisms, underlying a complex regulation of autophagy [113–117], micro-RNA [118–122], p53 [123–126], Bcl-2 [127, 128] family and interleukins [31, 32, 129, 130] able to also impinge on the above-mentioned metabolisms.

Therefore, the analysis followed considering correlation to clinical data, the analysis of the disease-free survival (DFS) of patients stratified according to the expression levels of some of the key genes involved in the serine metabolisms fails in showing statistically significant differences among the groups for PSAT1 (Fig. 4C) as well for SHMT1, SHMT2 and MTHFD1L where, anyhow, high expression shows a tendency to a worse disease course with higher number of progressions (Figs. 4D–F). Anyhow, grouping all the possible alterations in the genes involved in the serine metabolism stratifying patients between the altered and the unaltered group, there is a strong difference in terms of progression free survival with a better prognosis for the unaltered group (p < 0.001) (Fig. 4G), this might be regarded as the development of a potential prediction algorithm as already done in many other clinical contexts [131–134].

Apart from a possible role of the enzymes involved in the 1C metabolism as potential biomarker of progression in prostate cancer (specifically as a molecular signature) this metabolism might also represent a therapeutic option to be explored in this clinical context. The overall management of this cancer depends on a combined approach relying on surgery, radiotherapy, hormonal therapy or chemotherapy, depending on the status of the disease [135]. When chemotherapy is necessary, often in the case of tumors which show resistance to androgen deprivation therapy or inhibition of androgen synthesis, the use of microtubules synthesis inhibitors is the standard of care with drugs such as docetaxel or cabazitaxel [136]. Anyhow, resistance to these treatments, as well as resistance to targeted therapies (PARP inhibitors) can occur and potential alternative strategies are required. The inhibition of one-carbon metabolism, with an anti-folate therapy, might represent a significative option to be explored in a clinical context, and some data on the use of methotrexate, a known anti-folate drug, show efficacy in reducing prostate cancer progression in combination with luteinizing hormone releasing hormone, in cell lines end mouse xenograft models [137, 138]. The effect of the inhibition of the one carbon metabolism, should anyway be regarded with caution, since its impact on the immune system, inducing immunosuppression, might play a contradictory result promoting cancer progression [139].

## Conclusion

Despite being and extremely preliminary evaluation of the possible role of serine and one-carbon metabolism in prostate cancer, the present review shows that a pervasive alteration of these biochemical pathways is abundantly evoked during prostatic cancer pathogenesis and evolution. Anyhow, the analysis is quite partial due to the heterogeneity of the cohort of patients and the evaluation of the expression of genes at an RNA level rather than at a protein level. To address this specific issue, a multi-omics approach [140] might be beneficial for the dissection of complex metabolic scenarios like the one described. Of course, the generation of omics data and further analysis should rely on the developing machine learning algorithms [141, 142], allowing to detect molecular signatures among cancer specimens through the analysis of big data repositories.

Moreover, alteration at the levels of such a complex metabolism might also be exploited in order to develop biomarker for prediction or monitoring of the progression of the disease [77, 143, 144], as it is happening in the context of other types of cancer [145, 146].
